# Electricity in Surgery

**Published:** 1888-08

**Authors:** Donald Maclean

**Affiliations:** Prof. of Surgery


### SELECTIONS.

### From an Address on Surgery,

BY DONALD MACLEAN, M.D., PROF. OF SURGERY.

[Read before the Surgical Section at the recent meeting of the American Medical Association.]

### ELECTRICITY IN SURGERY.

Electricity is another modern addition to the armamentarium of the practical surgeon ; and although it has in some directions proved disappointing, still it can, I think, with truth be claimed that when used for suitable purposes, it has demonstrated its right to the respect and gratitude of the profession. Much harm has been done and much unfair prejudice excited against this useful agent by the absurd and extravagant claims which have from time to time been made in its behalf as the result of inaccurate observations or unscientific deductions, and there is reason to fear that this kind of obstruction to the proper recognition of electricity as a surgical agent has not yet entirely ceased. That certain forms and degrees of urethral and other strictures are capable of being remedied by electrolysis skillfully and carefully applied I am prepared to believe ; but when we are told that all other methods of treatment of this large and important class of surgical cases are to be completely superseded, or nearly so, by this one, my belief is that our duty as rational practitioners is to suspend judgment.

Cases of far-advanced, long-neglected stricture, with fistulæ in perineo, and large deposits of dense, inflammatory tissue, will, in my opinion, still furnish a useful field for the operation of external perineal urethrotomy, an operation which I have performed with the most satisfactory results in a large number of cases which I do not believe would have yielded to any other method of treatment. Some years ago I performed the old operation of urethrotomy without a guide in a case of complete occlusion of the urethra, the result of laceration from fracture of the pelvis. The patient had passed all his urine through a perineal fistula for five years, not a drop having passed by the natural channel in all that time. The occluded portion of the urethra extended to a length of more than two inches. The operation, in which I was assisted by my colleague, Prof. Frothingham, and my brother, Dr. A. C. Maclean, was a long and difficult one, but was crowned with *absolute success*. And surely it will not be claimed that either electricity or any other method of treatment could have held out the least shadow of hope in such a case.

The testimony of Apostoli Keith and others as to the efficacy of this agent in favorably affecting the growth and tendencies of uterine fibroid tumors, if confirmed by further experience, is certain to be regarded as marking a most important era in the history of this department of surgery.

In this connection the following very recent case appears to me sufficiently important and suggestive to give here in detail. Mrs. S. G., æt. 18, residing at Matamora, Ohio, was admitted to the University of Michigan Hospital, April 18, 1888. She is of German extraction. Her father died in 1875 of acute consumption. During his early life in Germany he appeared to have been in hospitals for treatment of tumors on arm and shoulder. During latter years he suffered very much from rheumatism occasionally. Seven brothers and sisters died in childhood in Germany ; cause of death unknown to the patient. The children born in America are healthy. Mrs. G. commenced to menstruate at twelve and has been regular since ; was married in June, 1887. During the fall of 1886 she first felt a lump in the left breast, which was painful. The swelling was about the size of a walnut and was movable. It increased very much during the winter and she suffered from shooting pains, which were more acute at her menstrual periods. On examination by me the tumor was found to occupy nearly the whole of the gland. It was hard, nodulated, the nipple retracted, and, in short, presented every appearance of scirrhus. Moreover a dense mass of considerable size existed in the axilla. Had this patient been a woman over thirty years of age I should undoubtedly have recommended and performed amputation of the breast.

The resemblance between this breast and many which I have amputated for cancerous disease, and which have been proved by ulterior investigation to be malignant, was very striking indeed. The age of the patient was the only consideration which led me to entertain a hope that the prognosis might not after all be so grave, and that the tumor might be amenable to treatment by milder means; and I determined before doing anything else to make a fair trial of electrolysis with it. For that purpose I requested my assistant, Dr. George A. Hendricks, to take charge of the treatment, which he did on April 19. The applications were made with positive electrode over the growths and negative over the sternum, the positive electrode being a circular copper plate one and a half inches in diameter, covered with fine sponge, the negative electrode a copper plate two inches square, covered with sponge. When in use the sponges were wet with a strong solution of salt and before application the parts were well washed with alcohol. On April 19 nine cells, seven miliamperes, were applied for ten minutes; on the 20th, thirty-eight cells, thirty miliamperes, for twelve minutes, applied over the tumor. Chloroform was given during these applications, as the patient could not tolerate them without anæsthesia. On April 21st, twelve cells, ten miliamperes, for ten minutes. On the same day the same application was made for the same length of time over the axillary tumor. The negative pole making the skin of the chest sore, a larger one was made, of an oval shape, five by eight, covered with fine sponge. On April 22nd, thirty-eight cells thirty miliaperes were applied for ten minutes over the breast and for the same length of time over the axillary tumor. By this time the breast tumor was found to be reduced to a spherical nodule about the size of a walnut. Contraction of the nipple had to a great extent disappeared. On April 24th, beginning with nine, increasing gradually to twenty-four cells, for twelve minutes on breast and axilla. On April 25th, 26th, and 27th, the same application; on April 28th, thirty cells; on April 29th, thirty-eight cells. After this the skin over the breast appeared somewhat irritated, but the tumor decidedly smaller. On April 30th, thirty-eight cells were applied over the breast and to the axilla. On May 3rd the patient was menstruating, and the treatment stopped for the present; but the growth by this time in the breast and in the axilla had almost entirely disappeared and the patient, considering herself cured, was most anxious to return to her home in Ohio, which she was permitted to do.

In chronic enlargement of the prostrate in old men I think I see in this direction a gleam of reasonable hope for a class of cases hitherto regarded as almost, if not quite, beyond remedy.

The value of electrolysis in the treatment of that common and important surgical condition known as nævus or aneurism by anastomosis is a triumph of surgery which belongs to our own generation. Previous methods of treatment, namely, excision, caustics, ligature, injection of astringents into the structure, are all inferior in every respect to the method by electrolysis. I have used and have seen used every one of these different methods, and have no hesitation in giving the preference to electrolysis on the score of safety, efficiency and ease of application. I here present for inspection a few photographs illustrative of a large number of cases of nævus, treated successfully by electrolysis at my public clinic.

